# Acute right-sided ischemic colitis in a COVID-19 patient: a case report and review of the literature

**DOI:** 10.1186/s13256-022-03276-z

**Published:** 2022-03-25

**Authors:** Hefzi Alratrout, Eric Debroux

**Affiliations:** 1 Department of General Surgery, King Fahd Hospital of the University, College of Medicine, Imam Abdulrahman Bin Faisal University, Dammam, Kingdom of Saudi Arabia; 2 Department of Digestive Surgery, Centre Hospitalier de l’Université de Montréal (CHUM), Montreal, QC Canada

**Keywords:** Case report, COVID-19, Right-sided ischemic colitis

## Abstract

**Introduction:**

In addition to attacking the respiratory system, the coronavirus disease may attack the gastrointestinal tract in various ways, one of which is by creating a coagulopathy that may lead to acute ischemia of the bowel, increasing morbidity and mortality rates in these patients.

**Presentation of case:**

We present a case of a white 72-year-old European male, who was admitted to the intensive care unit after developing COVID-19-induced acute respiratory distress syndrome. On the third week, despite a favorable evolution of his respiratory symptoms, the patient became clinically septic; laboratory findings showed an augmentation of his d-dimer, fibrinogen, C-reactive protein, and procalcitonin levels. Imaging showed signs of ischemia of the right colon. The patient was taken to the operating room; only the right side of his colon was ischemic, with a well demarcated cut-off. A laparoscopic right hemicolectomy with a terminal ileostomy was performed. The patient was able to go home 2 weeks after surgery.

**Discussion and conclusion:**

Ischemic colitis is an uncommon pathology in the general population, and is rare in COVID-19 patients. Most cases of ischemic colitis in COVID-19 patients in the literature were limited to the left colon, with < 10 cases involving the right colon. Accurate and quick diagnosis with appropriate management is the key to avoid any mortality in those patients who are already weakened by the coronavirus.

## Introduction

The World Health Organization (WHO) named it coronavirus disease 2019 (COVID-19), while the International Committee on Taxonomy of Viruses (ICTV) identified this virus as severe acute respiratory syndrome coronavirus 2 (SARS-CoV-2) [1, 2]. This viral infection has multifaceted presentations, ranging from asymptomatic to rapid multiple organ dysfunction syndrome. However, most patients present with cough, fever, and myalgia [3]. With time and experience, it has become evident that gastrointestinal (GI) symptoms can be present. In 2003–2004, during the outbreak of severe acute respiratory syndrome (SARS) caused by the severe acute respiratory syndrome coronavirus 1(SARS-COV-1), GI symptoms were similar to the current pandemic [4]. Acute ischemic colitis is a rare intestinal emergency that requires urgent and adequate management.

Coagulopathy due to COVID-19 has emerged as an important component of this infection, leading to significant morbidity and mortality. Infection by SARS-CoV-2 in severe cases can trigger a rapid and intense innate immune response (the cytokine storm) that leads to the release of proinflammatory and procoagulant cytokines (interleukins, tumor necrosis factor-alpha, interferons) [5], making those patients more susceptible to tissue damage and necrosis. Since the start of this pandemic, few cases of COVID-19-induced ischemic colitis have been reported in the literature [4–10]. We report an uncommon case of a 72-year-old male COVID-19 patient, who developed a well-limited and demarcated ischemic colitis of the right colon with a favorable recovery during hospitalization. We hope that this case and its management can be beneficial to medical and surgical colleagues in managing rare and uncommon COVID-19 GI complications. This work has been reported in line with the Surgical CAse REports (SCARE) and Preferred Reporting Of CasE Series in Surgery (PROCESS) criteria [11, 12].

## Case report

A 72-year-old European male with a medical history of arterial hypertension, type 2 diabetes mellitus, and end-stage renal failure on hemodialysis presented to our university hospital’s emergency department with fever and dyspnea for the last 24 hours. The patient was hemodynamically stable, and had an oxygen saturation of 90% at rest in room air. However, the patient quickly deteriorated in the emergency department, developing severe hypoxic respiratory failure that required intubation and mechanical ventilation. He was then admitted to the medical intensive care unit (ICU) as a case of COVID-19 acute respiratory distress syndrome (ARDS).

During the first 20 days in the ICU, the patient was evolving in a favorable manner, with supportive treatment, low doses of steroids, and on a prophylactic dose of subcutaneous low molecular weight heparin. The patient was being weaned gradually from mechanical ventilation until extubation on the 19th day. All his laboratory results during this period were within normal range. On the 21st day, the patient started to complain of abdominal pain and distention. He developed a fever around 39 °C with a tachycardia of 120 beats/min and no episodes of hypotension. His respiratory parameters were within the normal range without any signs of hypoperfusion. Clinical examination showed a generalized abdominal guarding.

His laboratory results were as follow: Hemoglobin: 117 g/L (reference: 130–170 g/L); white blood cells: 9.5 k/mm^3^ (reference: 4.0–10 k/mm^3^); platelets: 279 k/mm^3^ (reference: 130–400 k/mm^3^); lactic acid: 0.6 mmol/L (reference: 0.6–2.4 mmol/L); troponin: 0.04 ng/mL (reference: < 0.04 ng/mL); d-dimer: 1.0 μg/mL (reference: < 0.5 μg/mL); fibrinogen: 6.42 g/L (reference: 2–4.5 g/L); C-reactive protein: 361 mg/L (reference: < 10 mg/L); procalcitonin: 3.46 ng/mL (reference: < 2 ng/mL); international normalized ratio (INR): 1.06 (reference: 0.80–1.15); partial thromboplastin time: 26 seconds (reference: 22–31 seconds).

**Fig. 1:**
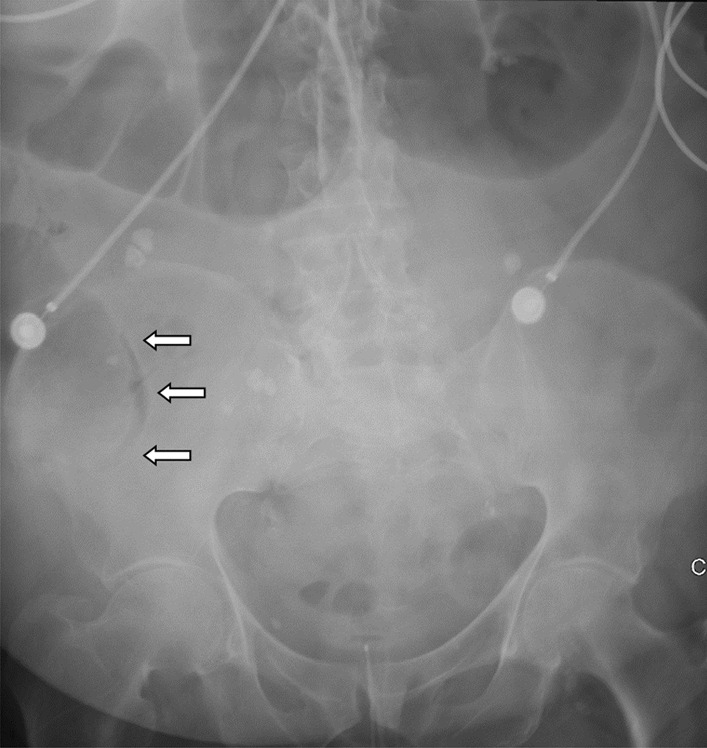
Abdominal X-ray showing pneumatosis intestinalis in the right colon (arrows)

**Fig. 2:**
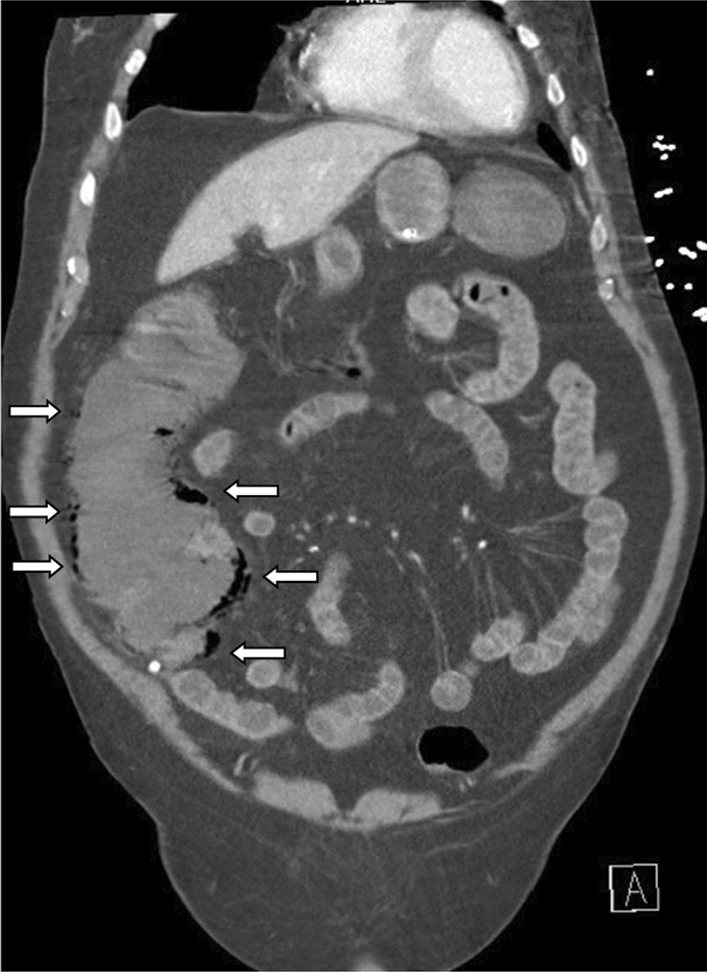
Abdominal computerized tomography (CT) scan with pneumatosis intestinalis of the right colon (arrows) (soft tissue window)

**Fig. 3:**
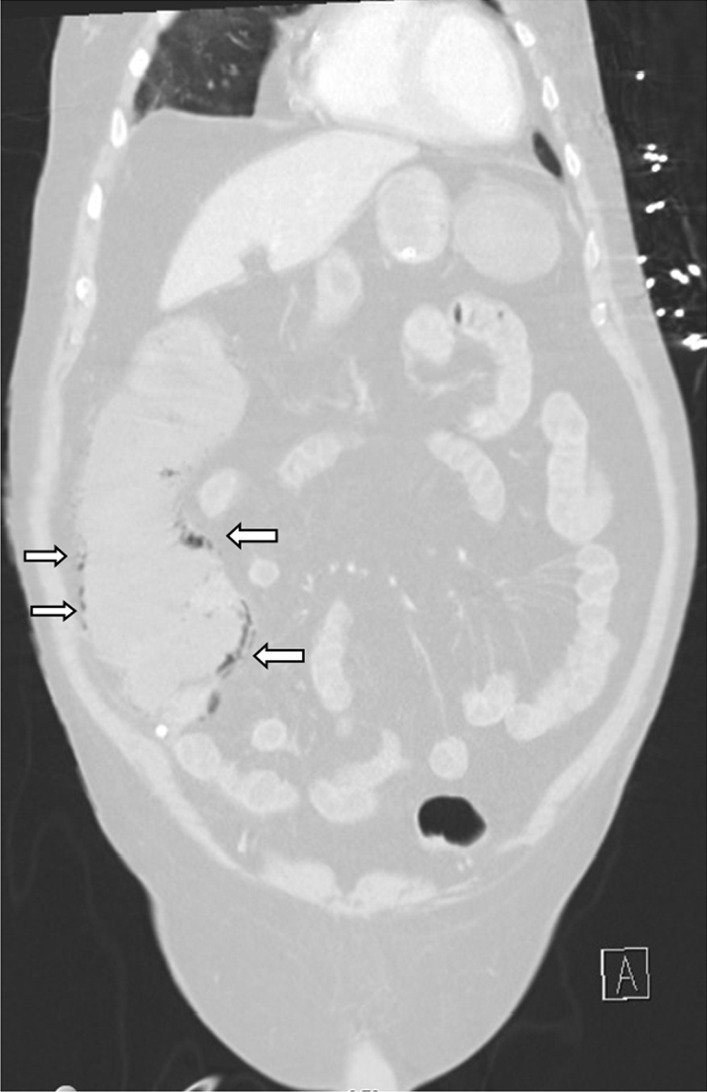
Abdominal computerized tomography (CT) scan with pneumatosis intestinalis of the right colon (arrows) (lung window)

An abdominal X-ray showed signs of pneumatosis intestinalis of the right colon (Fig. 1) and his abdominal computerized tomography (CT) scan with intravenous contrast showed signs of pneumatosis intestinalis of the entire right colon without signs of visceral perforation, and showed a normal nonobstructed superior mesenteric artery and vein in their proximal part (distal parts were poorly visualized on the CT scan due technical issues) (Figs. 2, 3).

**Fig. 4:**
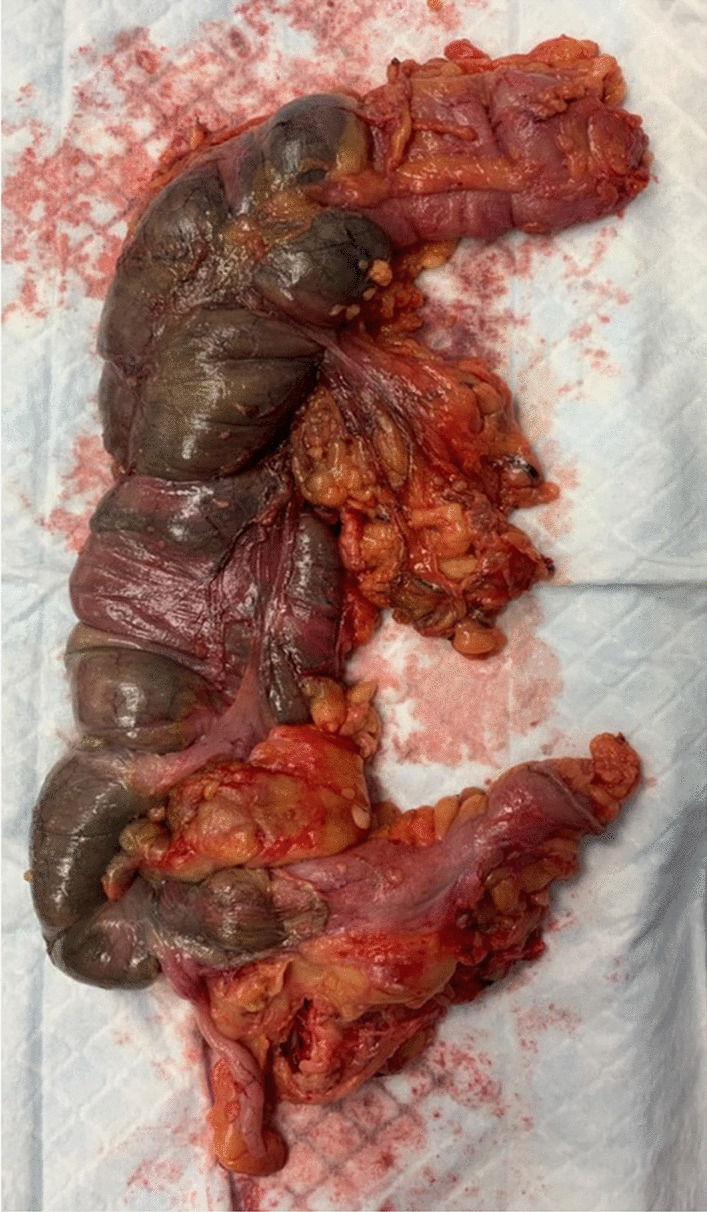
Gross specimen showing a well-demarcated ischemia of the right colon

The patient was taken to the operating room, where laparoscopy revealed a well-demarcated ischemia of the right colon. The distal part of ileocolic artery was thrombosed and the patient did not have a right colic artery. We proceeded with a right hemicolectomy with a terminal ileostomy (Fig. 4). The patient had a slow but favorable recovery, and was discharged from the hospital 2 weeks after surgery.

The final histopathology report concluded an acute ischemic colitis with transparietal necrosis and microvascular thrombosis, suggestive of COVID-19-related colitis.

The patient was readmitted to our unit 6 months later for an elective surgery, where he had has ileostomy taken down with a laparoscopic intracorporeal ileocolic anastomosis, with an excellent recovery, and was discharged home 4 days later.

## Discussion

Acute ischemic colitis is a medical emergency that can lead to high rates of morbidity and mortality if not dealt with in an adequate and prompt manner. Early diagnosis requires a high index of suspicion, with early imaging with or without endoscopy. Endoscopy can be used in diagnosing ischemic colitis. However, during the COVID-19 pandemic, this tool was usually abandoned due to the lack of personal protective equipment and the risk of viral transmission to health care workers. In our case, the clinical presentation of our patient with his CT scan results made the choice of going straight to the operating room without an endoscopy an easy one. Ischemic colitis is an uncommon condition where blood supply decreases to certain areas of the colon, leading to mucosal injury, then cellular ischemia and necrosis [13]. The “watershed” areas (splenic flexure and rectosigmoid junction) are most at risk of developing ischemia due to their limited collateral vascularization [14]. The etiologies of ischemic colitis are multiple, but mainly divided into nonocclusive (hypoperfusion) and occlusive (embolic or thrombotic) [15]. Angiotensin-converting enzyme 2 (ACE-2) is the target receptor for COVID-19, which is not only expressed in alveolar cells but also in the esophagus, gastric epithelial cells, small intestine, and colonic cells [16, 17]. Infection by SARS-CoV-2 in rare severe cases triggers an intense and rapid innate immune response (the cytokine storm) that leads to the release of proinflammatory and procoagulant cytokines (interleukins, tumor necrosis factor-alpha, interferons) [5]. With SARs-CoV-2 having a great affinity for the membrane receptors of ACE-2, this facilitates its entry into cells and its replication, which leads to cell death and the release of pathogen-associated molecular patterns (PAMPS) and damage-associated molecular patterns (DAMPs). Thus triggering a hemostatic and inflammatory response [18, 19], demonstrating the dangerous potential of this virus in invading the GI tract.

It has been well established that SARS-CoV-2 causes a hypercoagulability state, characterized by both microangiopathy and systemic coagulation defects, and that certain COVID-19 patients have high inflammatory markers predisposing them to vascular thrombosis [20]. With these two pathological elements, COVID-19 patients are at higher risk of developing ischemic colitis than other patients.

From the limited cases of GI ischemia in COVID-19 patients in the literature, almost half of patients died, even after surgical intervention [4–10]. This shows the extent of severity of this virus when attacking the GI system. To the best of our knowledge, this is the second case of right-sided ischemic colitis in a COVID-19 patient who survived. Almeida *et al*. [9] described two other cases involving the right side of the colon, unfortunately both patients died during hospitalization.

The coagulation profile and correlation among d-dimers, C-reactive protein levels, and COVID-19 severity has been stressed in the literature [21]. Most cases of coagulopathies seen in COVID-19 patients had a d-dimer level four times the normal value [6–8]. Others reported coagulopathies in their COVID-19 patients only when the d-dimer reached 14 times the normal level [9]. Yet in some cases, an increased level of d-dimer to twice that of normal was enough to trigger this coagulopathy cascade, in addition to the adjacent inflammatory storm [7]. This was the case in our patient, having a d-dimer level twice the normal value, and a very high level of C-reactive protein. We believe that the threshold needed to induce coagulopathy differs between patients, with other medical comorbidities playing a role in this pathology.

We acknowledge that our patient carried a risk of ischemic colitis, even before his infection by the coronavirus; because of his end-stage renal failure that requires hemodialysis, he is susceptible to hypoperfusion that may lead to ischemic colitis. Nevertheless, we strongly believe that his ischemic episode was directly related to the virus. Our patient was hemodynamically stable, with an excellent toleration of his hemodialysis sessions without hypotension episodes, precluding the possibility of a hypoperfusion-induced ischemia. Moreover, the fact that the ischemia involved the right colon, which is well vascularized and not a “watershed” area, further excludes the idea of a hypoperfusion state. In addition, our patient’s COVID-19-related inflammatory markers (fibrinogen, procalcitonin, and C-reactive protein) were remarkably high, and the onset of the ischemia during the third week was consistent with the period of hyperinflammation and cytokine storms, leading to a hypercoagulable state predisposing him to microvascular thrombosis, making the association between COVID-19 and his ischemic colitis more evident. All of this goes in parallel with the histopathology report of microvascular thrombosis.

## Conclusion

COVID-19 patients may well present with GI manifestations that can be challenging to manage, keeping in mind that ischemia, functional obstruction, and distention are possible complications of this virus. We hope that our case can add to the limited literature about the possibility of the hypercoagulable state leading to severe GI complications in COVID-19 patients.

## Data Availability

All supporting data and images are available at demand
